# Medical Miscellany

**Published:** 1919-05

**Authors:** 


### MEDICAL MISCELLANY

### War Risk Insurance Bureau Is in Charge of 28 Hospitals Devoted to the Work.

Washington, May 5.—An extensive program for caring for disabled soldiers after their discharge from military service was announced today by the War Risk Insurance Bureau, which is charged by Congress with this work. Twenty-one hospitals with a capacity of 1,500 beds already are in use, and the War Department has turned over to the Treasury seven camp hospitals for care of disability cases. These are to be enlarged and improved out of the $9,000,000 fund appropriated for hospitals for disabled soldiers, to be controlled by the War Risk Insurance Bureau and conducted by the Public Health Service, another Treasury agency.

When treatment in the hospitals of the War Department fails to restore men to such condition that they are fit for active service and they are discharged, the work of the Bureau of War Risk Insurance begins.

Under the provisions of the War Risk Insurance Act all soldiers who are 10 per cent disabled from causes suffered in the line of duty are entitled to compensation and to treatment by the Bureau of War Risk Insurance. Any man disabled in the service may take application for treatment to the Public Health Service station in his home town or at the nearest station.

Men who after discharge develop physical disabilities attributable to military service are entitled to treatment. The Bureau of War Risk Insurance may be called upon to care for more than 25,000 cases of men discharged for tubercular tendencies, it was announced.

The War Department has turned over to the Treasury Department for the care of disability cases hospitals located at Camp Cody, N. M.; Camp Hancock, Ga.; Camp Joseph E. Johnston, Fla.; Camp Logan, Texas; Camp Fremont, Cal., and at Perryville, Md. About $750,000 will be expended for enlarging and improving these hospitals.

The Treasury Department has purchased the Speedway Hospital at Chicago, with a capacity of 15,000 beds.

Another hospital to be taken over is located at Corpus Christi,

Texas, while $1,500,000 will be expended in the erection of a hospital at Dawson Springs, Ky., on land acquired as a gift to the Government.

A hospital costing $900,000 will be built at Norfolk, and $550,000 has been set aside for the erection of a hospital in or near the District of Columbia. The Marine Hospital at Stapleton, State Island, will be taken over.

---

**Military Hospitals Use Music Treatment.**

The use of music as a regular part of the treatment of the wounded soldiers, as distinguished from its general use as entertainment, is meeting with so much favor on the part of physicians and is being so generally adopted in the military hospitals of the United States that it has already become possible to formulate experience gained and draw some important general conclusions. One of these conclusions is that music is of extreme importance and valued in shell shock cases.

According to Dr. H. R. Humphries, who was consulting neurologist in the selective service headquarters of New York State, and who has made extensive observations of the treatment in Government hospitals of cases in his particular field, even the anticipation of a concert or a "sing" has a salutary effect on the men. It is best to have the patients themselves take an active part in the music, which may be either vocal or instrumental, but excellent results are also obtained from listening.

*Daily Concerts Advisable.*

"Music as such," says Doctor Humphries, "is proving so wonderfully helpful in the treatment of all nervous cases, especially war neurosis (shel shock) that concerts for those so afflicted should be held in the wards at least every day. Unless there are features counter-indicating, these concerts should take place at a regular hour.

"Merely looking forward to the music serves to quiet the patient's nerves and relieve his mind from introspection. During the time of the concert a highly desirable state of mental repose is attained. The attention of the men is centered on the melody and rhythm. This prevents them from thinking of their own troubles, which is always an important aim in the treatment of nerve disorders.

*Good Music Essential.*

"Only good music, and preferably that with a well-defined

### SHERMAN'S
### Bacterial Vaccines
### Efficient - Dependable

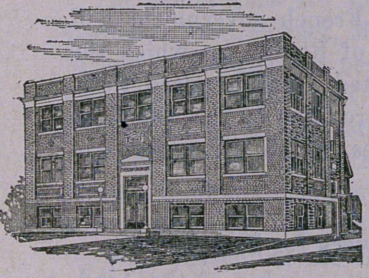

At this season of the year may we call your attention to the ever increasing use of Sherman's Vaccines in the prophylaxis and treatment of HAY FEVER.

DAILY USERS OF VACCINES USE SHERMAN'S

G. H. SHERMAN, M.D.
Detroit, Mich.
U.S.A.

WRITE FOR LITERATURE

melody, is suitable for this purpose. We find that even the men whose tastes ran to ragtime exclusively soon came to care much more for the other types.''

Doctor Humphries then cited a number of cases of men severely afflicted with war neurosis and extremely apathetic as a result who had previously played some instrument. Wherever these men have been induced to take up again the instrument to which they had been accustomed their recovery was hastened and made more complete.

---

**Bonus For Discharged Service Men and Nurses.**

The Red Cross is trying to spread the information regarding the provisions of the new Revenue Bill for a $60 bonus to be paid to all soldiers and nurses discharged since April 1, 1917.

As many soldiers were discharged prior to the passage of the bill, the War Department is anxious that information regarding the bonus should be disseminated as widely as possible. Those who are discharged hereafter will receive this bonus on the same roll or voucher upon which they are paid their final pay.

Employers, editors and all persons in a position to give this information are asked to cooperate with the War Department in reaching all discharged men and nurses. Red Cross chapters, branches and auxiliaries are request to post notices in prominent places.

---

### Memorial to American Dead.

Overlooking 15,000 roofless houses of Rheims, and overshadowed by its battered cathedral, will stand the first great memorial to the American dead in France. This is assured by official advices which reached Paris headquarters of the American fund for French wounded. The French government will need to find ground in the environs of Rheims on which the great American hospital will be erected. One hundred thousand dollars for this purpose will be provided by the American fund organization in the United States, which will proceed to raise the endowment for the hospital.

---

### Operation on Heart Was War Discovery for Fine Surgery.

Chicago, April 22.—Prof. O. Laurent, member of the Academy of Medicine of Paris, told the Chicago Medical society that operations on the heart, although now in the experimental stage, will before long be as common as ordinary surgical cases.

Professor Laurent based the prediction on successful operations in war hospitals and showed moving pictures of operations performed successfully on soldiers' hearts.

---

### Murder Defendant Fights for Standing In Medical Society.

Dr. B. Clark Hyde, tried three times on the charge of causing the death of Col. Thomas Swope, millionaire uncle of Mrs. Hyde, in 1911, has filed mandamus proceedings in the Circuit Court to compel the Jackson County Medical Society to reinstate him as a member.

Judge Robertson of the Circuit Court issued a temporary writ returnable March 31 ordering the society to show cause for the suspension or to reinstate Hyde.

---

RATHER HOPELESS:—"Doctor," asked the anxious young man, "is there anything that will prevent me from becoming totally bald at forty?"

"Why, yes. A fatal automobile accident might do it."

